# Protective effects of alpinumisoflavone against streptozotocin-induced gestational diabetes mellitus in female rats: mechanistic insights and therapeutic potential

**DOI:** 10.1590/acb401425

**Published:** 2025-10-17

**Authors:** Yan Zhu, Yu Zhang, Feiyan Liu, Jiao Xiao

**Affiliations:** 1 The First Hospital of Nanchang – Department of Endocrinology – Nanchang – Jiangxi – China.; 2 People’s Hospital of Xinchang – Department of Endocrinology – Xinchang – Zhejiang – China.; 3 First Affiliated Hospital of Nanchang University – Department of Haemodialysis – Nanchang – Jiangxi – China.; 4 The Affiliated Nanhua Hospital – Hengyang Medical School University of South China – Department of Endocrinology – Hunan – Hengyang – China.

**Keywords:** Diabetes, Gestational, Inflammation, Insulin

## Abstract

**Purpose:**

To examine the protective effect of alpinumisoflavone against streptozotocin (STZ)-induced gestational diabetes mellitus (GDM) in female rats and explored the underlying mechanisms.

**Methods:**

Female rats were used in this study, and intraperitoneal administration of STZ (55 mg/kg) was used to induce diabetes. Body weight, blood glucose level, fetuses, placental weight, and placental index were estimated. Oral glucose tolerance test (OGTT) and insulin tolerance test (ITT) were performed. The levels of resistin, glycated hemoglobin (HbA1c), hepatic glycogen, free fatty acid (FFA), adiponectin, serum C-peptide, leptin, visfatin, intercellular adhesion molecule 1 (ICAM-1), vascular cell adhesion molecule 1 (VCAM-1), lipids, oxidative stress, inflammatory cytokines, and other parameters were estimated. mRNA expression was estimated in the pancreatic tissue.

**Results:**

Alpinumisoflavone treatment significantly (*p* < 0.001) enhanced body weight and fetuses and decreased placental weight and placental index. Alpinumisoflavone treatment significantly (*p* < 0.001) decreased blood glucose levels (BGL) and improved plasma insulin levels. Alpinumisoflavone treatment significantly (*p* < 0.001) suppressed the glucose and insulin levels in the OGTT and ITT. Alpinumisoflavone treatment significantly (*p* < 0.001) altered the levels of resistin, HbA1c, hepatic glycogen, FFA, Adiponectin, serum C-peptide, leptin, visfatin, ICAM-1, and VCAM-1; lipid parameters; oxidative stress parameters; inflammatory cytokines and inflammatory parameters, viz., cyclooxygenase-2 (COX-2), and nuclear kappa B factor (NF-κB). Alpinumisoflavone treatment significantly (*p* < 0.001) altered the mRNA expression levels of Toll-like receptor 4, nuclear kappa B factor65 (NF-κB65), NOD-, LRR-, pyrin domain-containing protein 3 (NLRP3), MyD88, SREBP-1, stearoyl-CoA desaturase-1 (SCD1), FAS, and ACC.

**Conclusion:**

Alpinumisoflavone has a protective effect against STZ-induced GDM via alteration of the TLR4/MyD88/NF-κB signaling pathway.

## Introduction

Gestational diabetes mellitus (GDM) is a common metabolic complication characterized by inflammation, glucose intolerance, hyperinsulinemia, and oxidative stress, which are commonly enhanced during pregnancy^1^. Various risk factors, such as the age at obesity, heredity, and gestation, may contribute to the initiation of GDM^2^^,^ 3. GDM presents risks to both the mother and the offspring. Elevated glucose levels during pregnancy can adversely affect fetal development and increase the risk of complications during childbirth^4^^,^ 5. Additionally, women with GDM are at a higher risk of developing insulin resistance, which can lead to long-term health issues such as cardiovascular disease and type-2 diabetes^5^^,^ 6.

The incidence of GDM has gradually increased globally each year owing to lifestyle changes^6^. Normal pregnancy is linked to increased glucose levels and suppressed insulin sensitivity to store energy in the fetus; pregnancy is associated with the expansion of maternal insulin resistance^6^. During pregnancy, the body has a high demand for insulin, which further enhances the load on the pancreatic β-cells^4^^,^ 7. Due to the enhanced insulin needs during pregnancy, β-cells are enhanced in number and size to generate more insulin, which helps prevent hyperglycemia by balancing blood sugar levels^6^^,^ 8.

Dysfunctional proliferation of β-cells has been linked to the onset of hyperglycemia and maternal insulin insufficiency, subsequently leading to the development of GDM in mice^6^. Advancements in diabetes mellitus research have suggested that metabolic dysfunction induced by GDM during pregnancy may increase the risk of obesity and diabetes in the fetus at birth. The risk to offspring in mothers with GDM has been also linked to birth weight and maternal obesity^9^. Children observed to be large for gestational age at birth were more prone to obesity than those with a normal birth weight. Recently, it has been shown that low- to high-risk mothers can be identified by measuring fetal abdominal circumference^10^.

The exact pathophysiology of GDM has not been fully explored. However, various reports have shown that insulin-antagonistic hormones, such as estrogen, prolactin, and progesterone, are generated via the placenta and play a crucial role in the expansion of GDM^3^. In the early stages of pregnancy, the boosted inflammatory reaction and oxidative stress play crucial roles in the development of GDM and participate in the progression of hyperglycemia^3^^,^ 11. Chronic hyperglycemic conditions are directly related to inflammatory reactions, in which boosted cytokine levels further trigger the impairment of pancreatic β-cells^3^^,^ 12. Inflammatory regulators play a crucial role in GDM initiation. Tumor necrosis factor-α (TNF-α) also plays a crucial role in insulin production. Various cytokines play a significant role in β-cell apoptosis, and other mechanisms have been reported to impair β-cell apoptosis^3^.

It is well known that synthetic drug has side effects, and medicinal plants have a long history in the treatment of various diseases. Medicinal plants have been used for centuries to treat various ailments, including diabetes mellitus^13^. Many plants and their phytoconstituents (bioactive compounds) have shown promising effects in managing blood glucose levels, offering an alternative or complementary approach to conventional synthetic drugs^14^^,^ 15. Medicinal plants offer a promising alternative or complementary approach to managing diabetes mellitus. Their diverse mechanisms of action, combined with their long history of use, support their potential to control glucose levels and improve overall health^14^^,^ 16.

Alpinumisoflavone (C_20_H_16_O_5_) is a naturally occurring isoflavonoid, a type of flavonoid. It is found in various plants, particularly those belonging to the Fabaceae (legume) family^17^^,^ 18. Alpinumisoflavone is a promising natural compound with various biological activities and therapeutic potential. Alpinumisoflavone exhibits antioxidant effects by scavenging free radicals, thereby protecting cells from oxidative stress injury^19^^–^ 21. Alpinumisoflavone has various pharmacological properties, including antimicrobial, antioxidant, anti-inflammatory, and anticancer activities. The anti-inflammatory effect of alpinumisoflavone is evident from suppressed inflammatory markets in animals^20^^,^ 22.

With this substantiation, the synergistic effects of the antidiabetic and anti-inflammatory effects of alpinumisoflavone were tested in rodents that were induced by streptozotocin (STZ) to induce GDM. Furthermore, we estimated the protective effect of alpinumisoflavone on insulin sensitivity and suppressed oxidative stress that occurs via the TLR4/MyD88/NF-κB Signaling pathway.

## Methods

### Experimental animal

Swiss Wistar rats (weight: 200 ± 20 g; sex: male and female; aged: 10–12 weeks old) were used in this experimental model. The rats were housed in a departmental animal house and kept in an acrylic plastic cage under controlled conditions (temperature, 20 ± 5°C; 65–75% relative humidity; 12/12-h dark/light cycle). The rats received standard pellet food and tap water *ad libitum*.

All animal studies were performed in accordance with the Institutional Animal Care and Use Committee, and the procedures were strictly followed according to the guidelines of the committee. The current study was carried out in the Department of Endocrinology, Hengyang Medical School, University of South China, Hunan Hengyang 421002, China.

### Toxicant preparation

STZ was dissolved in citrate buffer (pH = 4.5)^23^^,^ 24, and nicotinamide was soluble in normal saline^3^.

### Experimental design

For pregnancy, the female and male rats were allowed to mate, and female rats lacking a pessary the next day were considered pregnant for day 0. The pregnant rats were divided into five groups, and each group contained six female rats, as follow:

- Group I: normal control;
- Group II: GDM control;
- Groups III: GDM + alpinumisoflavone (5 mg/kg);
- Groups IV: GDM + alpinumisoflavone (10 mg/kg);
- Groups V: GDM + alpinumisoflavone (20 mg/kg).

Intraperitoneal administration of STZ (55 mg/kg)^25^ and nicotinamide (110 mg/kg)^26^ has been used to induce diabetes mellitus in rats^3^. First, STZ was administered to the rats, and, after 15 minutes, nicotinamide was administered. Rats with a blood glucose level > 250 mg/dL were considered diabetic controls. The rats were orally administration of alpinumisoflavone (5, 10, and 20 mg/kg) for 20 days.

The body weights of rats in all groups were determined at different time intervals. Furthermore, fetuses, placental weight, and indices have been estimated^10^^,^ 25.

### Glucose tolerance test and insulin tolerance test

A previously reported method was used to estimate oral glucose tolerance with minor modifications^26^. The rats were fasted overnight (6 h), and each group received intraperitoneal glucose administration (2 mg/kg, body weight). Glucose levels were estimated in all rats at regular time intervals (0, 30, and 60 minutes) using a glucometer (Roche Diagnostics). For the insulin tolerance test, the rats were intraperitoneally administered insulin (1 mU/kg, body weight). After 1 h of fasting, the same procedure was carried out as in the glucose tolerance test^27^.

### Blood and tissue sample

At end of the study, the rats were fasted overnight (12 h) and anesthetized using the diethyl ether, and blood samples were collected via puncturing the retro orbital Plexure and centrifuged at 10,000 g rpm for 10 minutes to separate the serum. The serum samples were stored at -20°C for further biochemical parameter estimation. Diethyl ether and ketamine were used to scarify the rats via cervical dislocation, and the pancreatic tissue was collected for histopathological observation. The 5-μm tissue of the pancreas was collected and stored in paraffin. The stored tissues were stained with hematoxylin and eosin.

### Determination of serum resistin, HbA1c, hepatic glycogen and free fatty acid level

The levels of resistin, hepatic glycogen, HbA1c, and free fatty acids were estimated using the enzyme-linked immunosorbent assay (ELISA) method following the manufacturer’s instructions and a previously reported method with minor modifications^25^^,^ 28 ^,^ 29.

### Adiponectin, serum C-peptide, leptin and visfatin

The levels of leptin, serum C-peptide, adiponectin, and visfatin were analyzed using an ELISA kit following the manufacturer’s instructions (Nanjing Jiancheng Bioengineering Institute, Nanjing, China).

### ICAM-1 and VCAM-1

ELISA kits were used to estimate the levels of intercellular adhesion molecule-1 (ICAM-1) and vascular cell adhesion molecule-1 (VCAM-1) following the manufacturer’s instructions (Nanjing Jiancheng Bioengineering Institute, Nanjing, China).

### Lipid parameters

Lipid parameters, including total cholesterol (TC), high-density lipoprotein (HDL), and triglyceride (TG), were estimated using available kits (Sigma Aldrich, United States of America) following the manufacturer’s instructions. Low-density lipoprotein (LDL) and very-low-density lipoprotein (VLDL) levels were estimated using Eqs. 1 and 2:


$$LDL=TC-HDL\times \frac{TG}{5}$$ (1)

$$VLDL=\frac{TG}{5}$$ (2)

### Oxidative stress parameters

Oxidative stress parameters, such as catalase (CAT), malonaldehyde (MDA), gluthatione peroxidase (GPx), glutathione (GSH), and superoxide dismutase (SOD), were analyzed using commercially available kits following the manufacturer’s instructions (Bio-Engineering Co., Ltd. Wuhan, China).

### Cytokines and inflammatory parameters

The levels of cytokines, such as TNF-α, interleukin-1β (IL-1β), interleukin-2 (IL-2), interleukin-4 (IL-4), interleukin-6 (IL-6), interleukin-10 (IL-10), and inflammatory parameters, such as cyclooxygenase-2 (COX-2) and Nuclear kappa-B factor (NF-κB), were estimated using ELISA kits following the manufacturer’s instructions (Sigma Aldrich, United States of America).

### Quantitative reverse transcription polymerase chain reaction

Total RNA was isolated from the islet tissues using the manufacturer instructions of the RNA Mini kit (QIAGEN Gaithersburg, MD, United States of America), and reverse transcribed into cNDAs using the WuantiTect Reverse Transcription kit (Qiagen) to estimate the differential expression under the following conditions: 95°C for 10 minutes, 40 cycles of 95°C for 10 seconds, and 60°C for 60 seconds. The primers used in this study are listed in Table 1.

**Table 1:** List of primer and gens.

S. No	Gene	Primer sequence (5’-3’)	
		Reverse	Forward
1	TLR4	GGTGGCTTAGGCTCTGATATGC	CTGCAGGTGCTGGATTTATCC
2	NLRP3	CGCAGATCACACTCCTCAAA	TACGGCCGTCTACGTCTTCT-
3	NF-κB	CTGGTCCCGTGAAATACACC	CCCATCTTTGACAATCGTGC
4	MyD88	CTCCTGCTGCTGCTTCAAGAT	ACTGCTCGAGCTGCTTACCAA
5	SCD-1	CTTTGACGGCTGGGTGTTTG	TGCTGATCCCCACAATTCCC
6	SIRT1	ATGGGTTCTTCTAAACTTGG	TAGGCGGCTTGATGGTAA
7	SREBP-1	GCGTTTCTACCACTTCAGGTTTCA	CCCTGCGAAGTGCTCACAA
8	ACC	CACACAACTCCCAACATGGTG	ACACTGGCTGGCTGGACAG
9	GAPDH	GATGGTGATGGGTTTCCCGT	AGTGCCAGCCTCGTCTCATA

Source: Elaborated by the authors.

### Histopathology

At the end of the experimental study, the pancreas tissue was removed from the rats, and 5-µm tissue was cut and fixed in the hematoxylin and eosin, and finally observed the tissue under the light microscope.

### Statistical analysis

The results of the current study are presented as mean ± standard error. Statistical analysis was performed between the two groups and analyzed using Student’s t-test using GraphPad Prism 8 (GraphPad Inc., San Diego, CA, United States of America). One-way analysis of variance was used to analyze comparisons among multiple groups. Statistical significance was set at *p* < 0.05.

## Results

### Body weight, fetuses’ weight, placental weight and placental index

**Figure 1:**
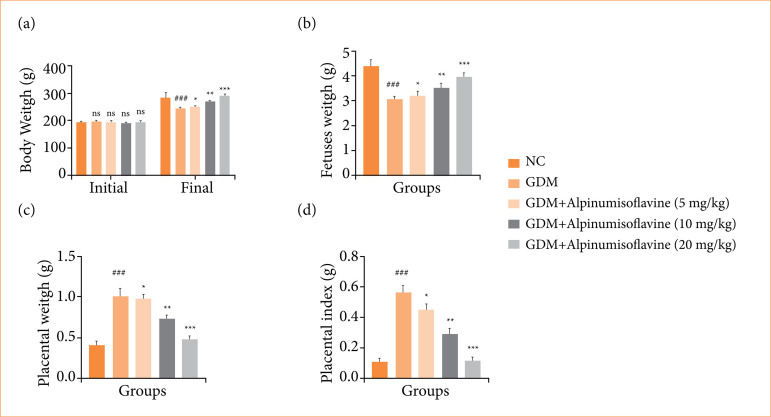
Effect of alpinumisoflavone on the **(a)** body weight, **(b)** fetuses’ weight, **(c)** placental weight and **(d)** placental index against streptozotocin (STZ) induced gestational diabetes mellitus (GDM) in rats. GDM control statistically analysis with normal control (###p < 0.001 as significant); tested group compared with GDM group (*p < 0.05, **p < 0.01 and ***p < 0.001 as significant).

Source: Elaborated by the authors.

Figure 1 shows the reduction in body weight of the GDM group rats. In this study, we found that almost the same body weight of female rats and GDM group rats exhibited a significant reduction in body weight (Fig. 1a), and alpinumisoflavone treatment significantly (*p* < 0.001) improved body weight. GDM group rats demonstrated reduced fetal weight (Fig. 1b) and boosted placental weight (Fig. 1c), and alpinumisoflavone significantly (*p* < 0.001) altered fetal and placental weight. Figure 1d shows the boosted placental index in the GDM group rats; alpinumisoflavone significantly (*p* < 0.001) decreased the placental index.

### Blood glucose level and plasma insulin level

**Figure 2:**
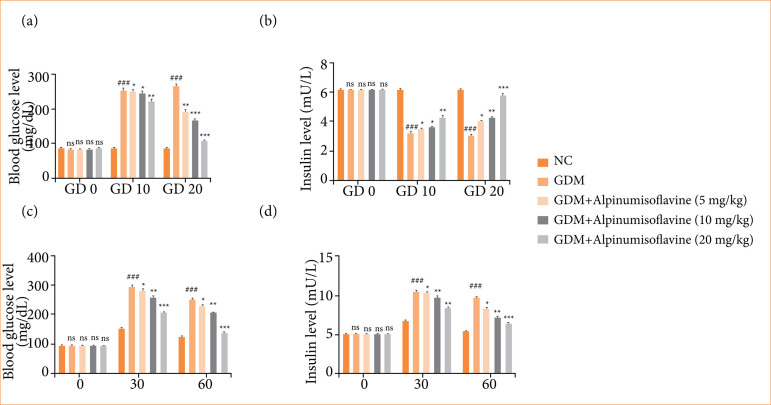
Effect of alpinumisoflavone on the **(a)** blood glucose level, **(b)** insulin level, **(c)** oral glucose tolerance test, and **(d)** insulin tolerance test against streptozotocin (STZ) induced gestational diabetes mellitus (GDM) in rats. GDM control statistically analysis with normal control (###*p* < 0.001 as significant); tested group compared with GDM group (*p < 0.05, **p < 0.01 and ***p < 0.001 as significant).

Source: Elaborated by the authors.

On GD0, the blood glucose level (BGL) of all rats was almost the same. However, on GD10, the BGL level of GDM group rats increased, and alpinumisoflavone treatment slightly reduced the glucose level. On GD20, the GDM group rats exhibited a significant (*p* < 0.001) increase in BGL, and alpinumisoflavone significantly (*p* < 0.001) suppressed the BGL level (Fig. 2a). Alpinumisoflavone (20 mg/kg)-treated rats exhibited the maximum reduction in BGL.

On GD0, the insulin levels of all rats were similar. However, on GD10, the insulin level of GDM group rats was reduced, and alpinumisoflavone treatment improved the insulin level. On GD20, the insulin level of GDM group rats was reduced, and alpinumisoflavone treatment significantly (*p* < 0.001) improved the insulin level (Fig. 2b).

### Oral glucose and insulin tolerance test

In the oral glucose tolerance test (OGTT), we administered glucose to the all-group rats, and, after 30 minutes, the glucose level of all group rats increased, and alpinumisoflavone treatment suppressed the glucose level. After 60 minutes, the glucose level of GDM group rats increased almost double the initial glucose level, and alpinumisoflavone treatment significantly (*p* < 0.001) suppressed the glucose level to almost the normal level (Fig. 2c).

A similar trend was observed in the insulin tolerance test; GDM group rats exhibited a significantly increased insulin level, and alpinumisoflavone treatment significantly (*p* < 0.001) reduced the insulin level (Fig. 2d).

### Resistin, HbA1c, hepatic glycogen and free fatty acid

**Figure 3:**
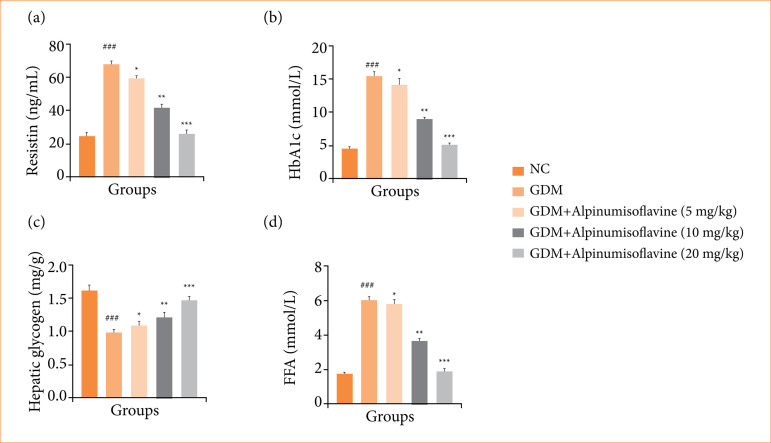
Effect of alpinumisoflavone on the **(a)** resistin, **(b)** glycated hemoglobin (HbA1c), **(c)** hepatic glycogen, and **(d)** free fatty acid (FFA) against streptozotocin (STZ) induced gestational diabetes mellitus (GDM) in rats. GDM control statistically analysis with normal control (###*p* < 0.001 as significant); tested group compared with GDM group (*p < 0.05, **p < 0.01 and ***p < 0.001 as significant).

Source: Elaborated by the authors.

GDM rats exhibited altered levels of resistin (Fig. 3a), HbA1c (Fig. 3b), hepatic glycogen (Fig. 3c), and free fatty acid (FFA) (Fig. 3d), and alpinumisoflavone treatment significantly (*p* < 0.001) restored resistin levels.

### Adiponectin, serum C-peptide, Leptin and visfatin

**Figure 4:**
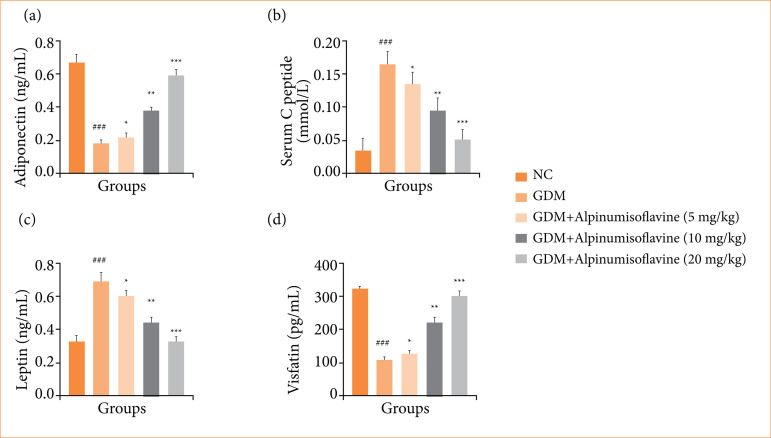
Effect of alpinumisoflavone on the **(a)** adiponectin, **(b)** serum C-peptide, **(c)** leptin, and **(d)** visfatin against streptozotocin (STZ) induced gestational diabetes mellitus (GDM) in rats. GDM control statistically analysis with normal control (###*p* < 0.001 as significant); tested group compared with GDM group (*p < 0.05, **p < 0.01 and ***p < 0.001 as significant).

Source: Elaborated by the authors.

GDM group rats exhibited modulated levels of adiponectin (Fig. 4a), serum C-peptide (Fig. 4b), leptin (Fig. 4c), and visfatin (Fig. 4d), and alpinumisoflavone treatment significantly (*p* < 0.001) altered the level to almost normal levels.

### ICAM-1 and VCAM-1

**Figure 5:**
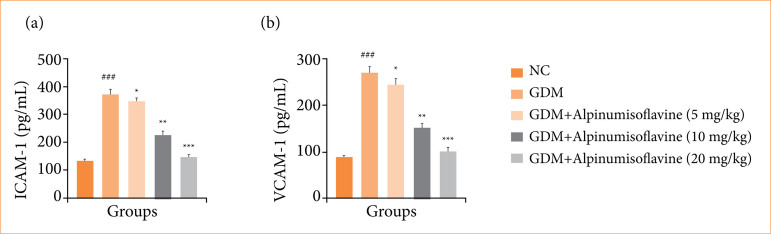
Effect of alpinumisoflavone on the **(a)** intercellular adhesion molecule-1 (ICAM-1) and **(b)** vascular cell adhesion molecule-1 (VCAM-1) against streptozotocin (STZ) induced gestational diabetes mellitus (GDM) in rats. GDM control statistically analysis with normal control (###p < 0.001 as significant); tested group compared with GDM group (*p < 0.05, **p < 0.01 and ***p < 0.001 as significant).

Source: Elaborated by the authors.

The levels of ICAM-1 (Fig. 5a) and VCAM-1 (Fig. 5b) increased in GDM group rats, and alpinumisoflavone treatment significantly (*p* < 0.001) suppressed the levels of ICAM-1 and VCAM-1.

### Lipid parameters

**Figure 6:**
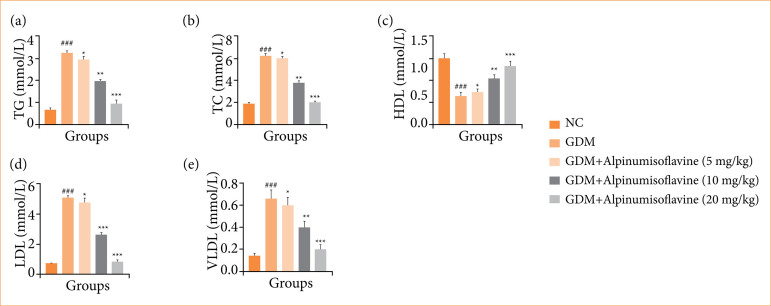
Effect of alpinumisoflavone on the **(a)** triglyceride (TG), **(b)** total cholesterol (TC), **(c)** high-density lipoprotein (HDL), **(d)** low-density lipoprotein (LDL), and **(e)** very-low-density lipoprotein (VLDL) against streptozotocin (STZ) induced gestational diabetes mellitus (GDM) in rats. GDM control statistically analysis with normal control (###*p* < 0.001 as significant); tested group compared with GDM group (*p < 0.05, **p < 0.01 and ***p < 0.001 as significant).

Source: Elaborated by the authors.

GDM group rats showed altered levels of lipid parameters such as TG (Fig. 6a), TC (Fig. 6b), HDL (Fig. 6c), LDL (Fig. 6d), and VLDL (Fig. 6e), and alpinumisoflavone treatment significantly (*p* < 0.001) restored the levels of lipid parameters.

### Oxidative stress parameters

**Figure 7:**
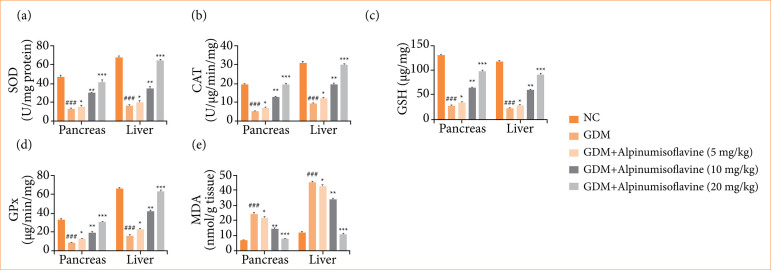
Effect of alpinumisoflavone on the **(a)** superoxide dismutase (SOD), **(b)** catalase (CAT), **(c)** glutathione (GSH), **(d)** gluthatione peroxidase (GPx), and **(e)** malonaldehyde (MDA) against streptozotocin (STZ) induced gestational diabetes mellitus (GDM) in rats. GDM control statistically analysis with normal control (###*p* < 0.001 as significant); tested group compared with GDM group (*p < 0.05, **p < 0.01 and ***p < 0.001 as significant).

Source: Elaborated by the authors.

In this study, we examined the oxidative stress parameters in the pancreas and hepatic tissue. GDM group rats showed reduced levels of SOD (Fig. 7a), CAT (Fig. 7b), GSH (Fig. 7c), and GPx (Fig. 7d) and increased MDA levels (Fig. 7e). Alpinumisoflavone treatment significantly (*p* < 0.001) altered the level of oxidative stress parameters.

### Cytokines and inflammatory parameters

**Figure 8:**
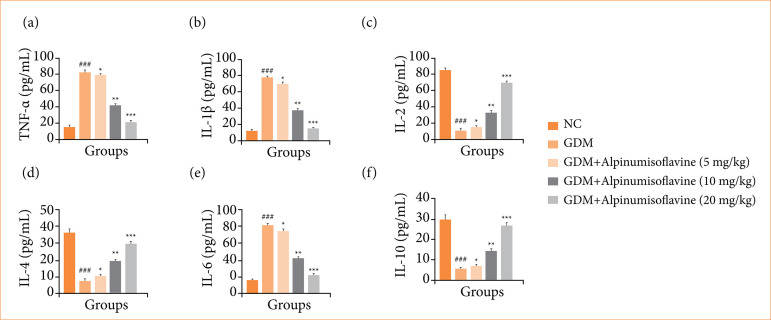
Effect of alpinumisoflavone on the **(a)** tumor necrosis factor-α (TNF-α), **(b)** interleukin-1β (IL-1β), **(c)** interleukin-2 (IL-2), **(d)** interleukin-4 (IL-4), **(e)** interleukin-6 (IL-6), and **(f)** interleukin-10 (IL-10) against streptozotocin (STZ) induced gestational diabetes mellitus (GDM) in rats. GDM control statistically analysis with normal control (###*p* < 0.001 as significant); tested group compared with GDM group (*p < 0.05, **p < 0.01 and ***p < 0.001 as significant)

Source: Elaborated by the authors.

GDM induced rats exhibited the levels of TNF-α (Fig. 8a), IL-1β (Fig. 8b), IL-2 (Fig. 8c), IL-4 (Fig. 8d), IL-6 (Fig. 8e), and IL-10 (Fig. 8f), and alpinumisoflavone treatment significantly (*p* < 0.001) restored the levels of cytokines.

**Figure 9:**
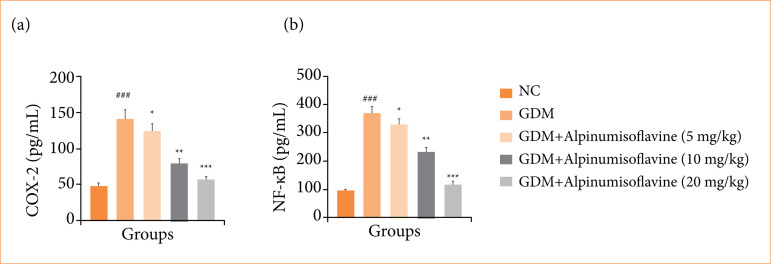
Effect of alpinumisoflavone on the **(a)** cyclooxygenase-2 (COX-2), and **(b)** nuclear kappa-B factor (NF-κB) against streptozotocin (STZ) induced gestational diabetes mellitus (GDM) in rats. GDM control statistically analysis with normal control (###*p* < 0.001 as significant); tested group compared with GDM group (*p < 0.05, **p < 0.01 and ***p < 0.001 as significant).

Source: Elaborated by the authors.

GDM-induced rats showed increased levels of COX-2 (Fig. 9a) and NF-κB (Fig. 9b), and alpinumisoflavone treatment significantly (*p* < 0.001) suppressed the levels of inflammatory parameters.

### mRNA expression

**Figure 10:**
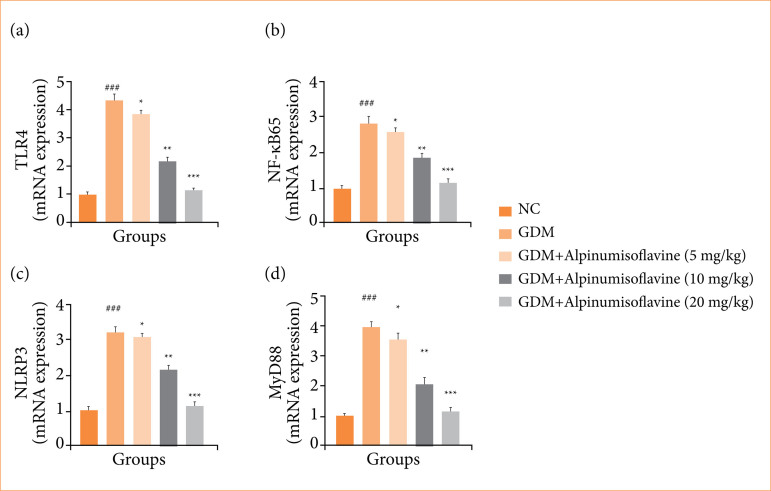
Effect of alpinumisoflavone on the **(a)** TLR4, **(b)** nuclear kappa B factor65 (NF-κB65), **(c)** pyrin domain-containing protein 3 (NLRP3), and **(d)** MyD88 against streptozotocin (STZ) induced gestational diabetes mellitus (GDM) in rats. GDM control statistically analysis with normal control (###p < 0.001 as significant); tested group compared with GDM group (*p < 0.05, **p < 0.01 and ***p < 0.001 as significant).

Source: Elaborated by the authors.

GDM-induced rats showed increased mRNA expression of Toll-like receptor 4 (TRL4) (Fig. 10a), nuclear kappa-B factor65 (NF-κB65) (Fig. 10b), pyrin domain-containing protein 3 (NLRP3) (Fig. 10c), and MyD88 (Fig. 10d), while alpinumisoflavone treatment significantly (*p* < 0.001) reduced mRNA expression.

**Figure 11:**
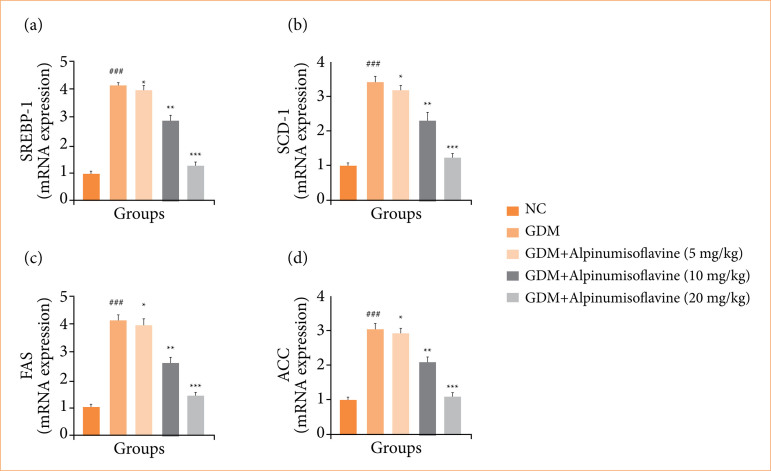
Effect of alpinumisoflavone on the **(a)** SREBP-1, **(b)** stearoyl-CoA desaturase-1 (SCD1), **(c)** FAS, and **(d)** ACC against streptozotocin (STZ) induced gestational diabetes mellitus (GDM) in rats. GDM control statistically analysis with normal control (###p < 0.001 as significant); tested group compared with GDM group (*p < 0.05, **p < 0.01 and ***p < 0.001 as significant).

Source: Elaborated by the authors.

GDM-induced rats exhibited enhanced mRNA expression of SREBP-1 (Fig. 11a), stearoyl-CoA desaturase-1 (SCD1) (Fig. 11b), FAS (Fig. 11c), ACC (Fig. 11d), and alpinumisoflavone treatment significantly (*p* < 0.001) reduced mRNA expression.

### Histopathology

**Figure 12:**
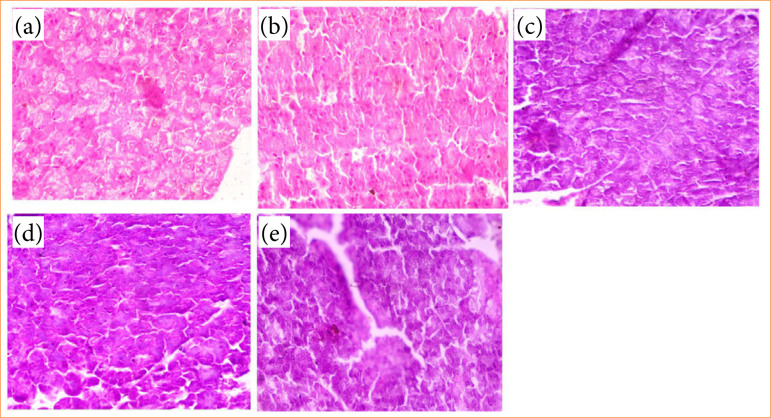
Effect of alpinumisoflavone on the histopathology. **(a)** Normal control, **(b)** streptozotocin (STZ) control, **(c)** STZ + alpinumisoflavone (5 mg/kg), **(d)** STZ + alpinumisoflavone (10 mg/kg) and **(e)** STZ+ alpinumisoflavone (15 mg/kg) against STZ induced gestational diabetes mellitus in rats.

Source: Elaborated by the authors.

In the manuscript, the histopathological examination of the pancreas from GDM-induced rats revealed significant destruction of pancreatic β-cells, a hallmark of diabetes progression. The administration of alpinumisoflavone showed a protective effect, as evidenced by the regeneration of pancreatic β-cells. This finding highlights the potential therapeutic role of alpinumisoflavone in restoring β-cell integrity and function, suggesting its ability to counteract the damage caused by GDM. The observed regenerative effect may be linked to the compound’s anti-inflammatory and antioxidant properties, which help mitigate oxidative stress and inflammation, key contributors to β-cell destruction (Fig. 12).

## Discussion

It is well known that GDM is a highly prevalent issue during pregnancy^7^. Reports suggest that 14.8% of the overall population of China is affected annually by GDM^30^. Insulin resistance is a major health concern in individuals with diabetes. If the body cannot compensate for this resistance during pregnancy, GDM can develop. The underlying pathogenic features of GDM, such as insulin resistance and compensatory increase in islet cell secretion, are similar to those observed in type-II diabetes mellitus. Long-term hyperglycemia exacerbates islet cell death and further impairs islet cell function, leading to various cellular responses such as inflammation and oxidative stress, thus creating a vicious cycle^10^. Novel diabetes medications play a crucial role in preventing injury induced by enhanced glucose levels in islet cells.

In GDM, the physician aimed to maintain the glucose level during the glycemic condition. A specific diet is essential for managing GDM; however, if dietary changes are insufficient, patients may require insulin therapy^10^. The initiation of insulin administration in GDM cases is still debated. Therefore, researchers are currently exploring various strategies to reduce the risks to both infants and mothers^7^.

GDM affects glucose metabolism during pregnancy and has several key effects. GDM typically results in elevated blood glucose levels (hyperglycemia) in mothers. This occurs because the body’s insulin sensitivity is reduced during pregnancy, especially in later stages^25^. In response to elevated blood glucose levels, the pancreas increases its insulin production to maintain normal glucose levels. However, the insulin response may be insufficient in GDM, leading to persistent hyperglycemia^12^.

OGTT is commonly used to diagnose GDM. In this test, a pregnant woman drinks a glucose solution, and blood glucose levels are measured at specific intervals. In women with GDM, glucose levels are higher and may not return to normal as quickly as in women without GDM^3^. The insulin tolerance test (ITT) measures how well the body responds to insulin by administering insulin and monitoring glucose levels over time.

In GDM, there may be reduced sensitivity to insulin, leading to higher glucose levels following insulin administration than in women without GDM^31^. GDM leads to elevated glucose levels owing to decreased insulin sensitivity during pregnancy. This condition is diagnosed using tests such as the OGTT, which shows impaired glucose tolerance, and ITT, which may demonstrate reduced responsiveness to insulin. The management of GDM typically involves dietary changes, exercise, and sometimes insulin therapy to control blood glucose levels and minimize risks to both the mother and baby^12^^,^ 25.

Several factors play a significant role in the pathophysiology and treatment of GDM. Resistin is a hormone secreted by adipose tissue (fat cells) that has been implicated in insulin resistance. Resistin levels may be elevated in individuals with GDM, contributing to decreased insulin sensitivity. This hormone can interfere with insulin action in peripheral tissues, such as muscle and adipose tissue, potentially exacerbating glucose intolerance^7^.

HbA1c reflects the average blood glucose level over the past two or three months. In the context of GDM, monitoring HbA1c levels can provide insight into long-term glucose control. Elevated HbA1c levels indicate poor glycemic control and may necessitate adjustments in treatment to reduce the risk of complications in both the mother and baby. Hepatic glycogen refers to the glycogen stored in the liver, which plays a crucial role in maintaining blood glucose levels^29^^,^ 31.

In GDM, impaired insulin sensitivity can disrupt normal regulation of hepatic glycogen storage and release. This can lead to fluctuations in blood glucose levels and contribute to hyperglycemia. Elevated levels of FAA, especially in conditions such as obesity or insulin resistance, can exacerbate insulin resistance and impair glucose metabolism^7^. In GDM, increased circulating FFA levels may contribute to the development or worsening of insulin resistance, thereby affecting glucose control.

Adiponectin plays a crucial role in insulin sensitivity. Higher levels of adiponectin are associated with improved insulin sensitivity and a lower risk of type-2 diabetes. Adiponectin levels may be reduced in GDM, contributing to insulin resistance. Low adiponectin levels have been observed in women with GDM and may exacerbate glucose intolerance during pregnancy.

Serum C-peptide level is a marker of insulin secretion. It is released in equimolar amounts along with insulin during the cleavage of proinsulin into insulin^29^^,^ 32. In GDM, serum C-peptide levels may be elevated because of increased insulin production by the pancreas in response to insulin resistance. Elevated C-peptide levels reflect an increased demand for insulin to maintain glucose homeostasis in the presence of reduced insulin sensitivity.

Leptin is another adipokine that regulates the energy balance and appetite. It is involved in satiety signaling and energy expenditure. Leptin levels can be elevated in GDM, especially in cases of maternal obesity. Elevated leptin levels are associated with insulin resistance and may contribute to metabolic disturbances observed in GDM. Visfatin is an adipokine with insulin-mimetic effects and is involved in glucose metabolism. Visfatin levels may be altered in GDM, although research on its specific role in GDM is less established compared to other adipokines. Some studies suggest that visfatin levels may be elevated in GDM, potentially influencing insulin sensitivity and glucose metabolism^7^.

The elevated levels of ICAM-1 and VCAM-1 observed in GDM suggest that inflammation and endothelial dysfunction are prominent features of this condition. These factors contribute to insulin resistance by impairing insulin signaling pathways and promoting chronic low-grade inflammation^25^. They also play a role in the pathogenesis of the vascular complications associated with diabetes, including GDM.

ICAM-1 and VCAM-1 are cell adhesion molecules that play significant roles in inflammation and endothelial dysfunction, both of which are linked to GDM pathophysiology. ICAM-1 is expressed on the surface of endothelial and immune cells. It facilitates the adhesion and migration of immune cells to the sites of inflammation. In GDM, elevated levels of ICAM-1 were observed. This suggests increased endothelial activation and inflammation, which are characteristic features of insulin resistance and GDM25. VCAM-1 is another adhesion molecule found in endothelial cells of blood vessels. It plays a key role in the adhesion and recruitment of leukocytes (white blood cells) to inflammation sites. Similar to ICAM-1, VCAM-1 levels are elevated in conditions associated with insulin resistance and GDM. Increased VCAM-1 expression indicates endothelial dysfunction, which is closely linked to metabolic disturbances and insulin resistance in GDM^7^.

Antioxidant parameters play a crucial role in GDM because of their ability to counteract oxidative stress, which is often elevated in individuals with diabetes, including those with GDM. GDM is associated with increased oxidative stress, which occurs when there is an imbalance between the production of reactive oxygen species (ROS) and the body’s ability to detoxify or repair the resulting damage. High blood glucose levels in GDM can promote ROS production through several mechanisms including mitochondrial dysfunction, activation of inflammatory pathways, and increased glucose oxidation^3^. The body has several antioxidant defense mechanisms that neutralize ROS and mitigate oxidative stress. Antioxidants help to protect cells and tissues from oxidative damage caused by elevated glucose levels in GDM. This protection is particularly important because oxidative stress can lead to endothelial dysfunction, inflammation, and tissue damage, which contribute to complications such as preeclampsia and macrosomia (high birth weight)^7^^,^ 25.

In addition, some antioxidants have been shown to improve insulin sensitivity in animal and human studies. By reducing oxidative stress, antioxidants may enhance insulin signaling pathways, thereby improving glucose metabolism in GDM. Antioxidants play an important role in fetal development and growth. Oxidative stress in GDM can affect placental function and fetal nutrient transport. Antioxidants help mitigate these effects, potentially reducing the risk of adverse outcomes in the baby. Several studies have explored the potential benefits of antioxidant supplementation in GDM^3^.

In GDM, cytokines play a significant role in the inflammatory response and immune regulation, thereby influencing both maternal and fetal health. TNF-α is a proinflammatory cytokine involved in inflammation and immune response regulation. Elevated TNF-α levels have been observed in women with GDM. It contributes to insulin resistance by impairing insulin signaling pathways in peripheral tissues and the liver^10^. TNF-α also promotes inflammatory responses that can affect placental function and contribute to complications, such as preeclampsia, and IL-1β is another pro-inflammatory cytokine that mediates immune responses and inflammation.

Increased IL-1β levels are associated with inflammation and insulin resistance. IL-1β can impair pancreatic β-cell function, leading to reduced insulin secretion^10^^,^ 33. It also contributes to endothelial dysfunction and may play a role in GDM pathogenesis. IL-2 is a cytokine involved in the regulation of immune responses, particularly T cell activation and proliferation. IL-2 levels may be altered in GDM, reflecting changes in immune function and potentially influencing maternal immune tolerance of the fetus. IL-4 is an anti-inflammatory cytokine that promotes Th2-type immune response and tissue repair. IL-4 levels may be dysregulated in GDM, affecting the balance between the pro-inflammatory and anti-inflammatory responses. Its role in GDM pathophysiology is less well studied than that of other cytokines^25^. IL-6 is a multifunctional cytokine that is involved in inflammation, immune response modulation, and metabolic regulation. Elevated IL-6 levels are commonly observed in GDM patients. IL-6 contributes to insulin resistance by promoting hepatic glucose production and impairing insulin signaling in peripheral tissues. It also plays a role in systemic inflammation and may influence placental function and fetal development. IL-10 is an anti-inflammatory cytokine that suppresses immune response and inflammation. IL-10 levels may be altered in GDM, potentially reflecting the body’s attempts to counteract excessive inflammation and immune activation. Dysregulation of cytokines in GDM reflects an imbalance between pro-inflammatory and anti-inflammatory responses, contributing to insulin resistance, inflammation, and potential complications. Monitoring cytokine levels and understanding their roles may help develop targeted interventions to manage GDM and improve maternal and fetal outcomes.

Several molecular pathways and proteins play significant roles in the pathophysiology and metabolic changes associated with the condition. TLR4 is a receptor that is involved in innate immunity and inflammation. It recognizes pathogen-associated molecular patterns (PAMPs) and damage-associated molecular patterns (DAMPs), thereby triggering inflammatory responses. Elevated TLR4 expression and activation have been observed in GDM patients. TLR4 activation can lead to increased production of pro-inflammatory cytokines (such as TNF-α and IL-1β) and contribute to insulin resistance and inflammation in GDM^7^. NF-κB is a transcription factor that regulates the expression of genes involved in inflammation, immune response, and cell survival. NF-κB activation is commonly increased in GDM, leading to the elevated expression of pro-inflammatory cytokines and inflammatory mediators. This contributes to insulin resistance and systemic inflammation observed in patients with GDM. NLRP3 is a part of the inflammasome complex which regulates the activation of pro-inflammatory cytokines IL-1β and IL-18. Activation of the NLRP3 inflammasome and increased IL-1β production have been implicated in the pathogenesis of GDM, contributing to inflammation, insulin resistance, and potentially adverse pregnancy outcomes^34^^,^ 35. MyD88 is an adaptor protein involved in signaling pathways downstream of TLRs, leading to NF-κB activation and cytokine production. MyD88-mediated signaling pathways contribute to the inflammatory response observed in GDM by linking TLR activation to NF-κB activation and subsequent cytokine release. SREBP-1 is a transcription factor that regulates the expression of genes that are involved in lipid synthesis and metabolism. Increased SREBP-1 activity has been observed in GDM, which promotes lipogenesis and contributes to dyslipidemia and insulin resistance^27^^,^ 34 ^–^ 36. SCD-1 is involved in the synthesis of monounsaturated fatty acids from saturated fatty acids. Elevated SCD-1 expression and activity have been associated with increased lipid accumulation and insulin resistance in GDM. FAS is an enzyme responsible for catalyzing fatty acid synthesis. Increased FAS expression and activity contribute to excessive lipid accumulation and may exacerbate insulin resistance in GDM^37^^,^ 38. ACC is an enzyme involved in fatty acid synthesis that catalyzes the carboxylation of acetyl-CoA to malonyl-CoA. Dysregulation of ACC activity can lead to increased fatty acid synthesis and lipid accumulation, contributing to metabolic disturbances observed in GDM. These molecular factors TLR4, NF-κB, NLRP3, MyD88, SREBP-1, SCD-1, FAS and ACC contribute to the pathophysiology of GDM by promoting inflammation, insulin resistance, dyslipidemia, and metabolic dysfunction. Understanding their roles can provide insights into potential therapeutic targets for managing GDM and reducing associated maternal and fetal complications^7^.

## Conclusion

The results of this study demonstrate the protective effect of alpinumisoflavone against STZ-induced GDM in female rats. Alpinumisoflavone treatment significantly improved several key parameters, including enhanced body weight, fetal weight, placental weight, and placental index. It effectively decreased BGL and improved plasma insulin levels, indicating an overall positive effect on the glucose metabolism.

The study also showed that alpinumisoflavone significantly suppressed glucose and insulin levels in both OGTT and ITT. Additionally, alpinumisoflavone treatment altered the levels of various biomarkers associated with GDM, including resistin, HbA1c, hepatic glycogen, FFA, adiponectin, serum C-peptide, leptin, visfatin, ICAM-1, VCAM-1, and lipid parameters (TG, TC, LDL, VLDL, and HDL).

Furthermore, oxidative stress markers (SOD, CAT, GSH, GPx, and MDA) and inflammatory cytokines (TNF-α, IL-1β, IL-2, IL-4, IL-6, and IL-10) were significantly modulated by alpinumisoflavone treatment. The expression of inflammatory parameters, such as COX-2 and NF-κB, as well as mRNA expression levels of TLR4, NF-κB65, NLRP3, MyD88, SREBP-1, SCD-1, FAS, and ACC, were also significantly altered.

These findings suggest that alpinumisoflavone exerts its protective effects against GDM by modulating the TLR4/MyD88/NF-κB signaling pathway. This study highlights the potential of alpinumisoflavone as a therapeutic agent for GDM and provides a foundation for further research and development in this area. In-depth studies focusing on the specific interactions of alpinumisoflavone with the TLR4/MyD88/NF-κB signaling pathway and other related pathways will further elucidate its anti-inflammatory and metabolic regulatory effects. Exploring the potential of alpinumisoflavone as part of a combination therapy with existing GDM treatments could provide synergistic effects and improve therapeutic outcomes.

## Data Availability

The data will be available of the request to the corresponding author.
